# Effectiveness and Complications of Bone Marrow Aspirate Concentrate in Patients with Knee Osteoarthritis of Kellgren–Lawrence Grades II–III

**DOI:** 10.3390/medicina60060977

**Published:** 2024-06-13

**Authors:** Ji-Hoon Baek, Su Chan Lee, Dong Nyoung Lee, Hye Sun Ahn, Chang Hyun Nam

**Affiliations:** Joint & Arthritis Research, Department of Orthopaedic Surgery, Himchan Hospital, Seoul 07999, Republic of Korea; jihoon011@naver.com (J.-H.B.); himchanhospital@naver.com (S.C.L.); fretless@hanmail.net (D.N.L.); ahs0614@naver.com (H.S.A.)

**Keywords:** effectiveness, complications, bone marrow aspirate concentrate

## Abstract

This study aimed to identify the effectiveness and potential complications on the harvest site and knee of bone marrow aspirate concentrate (BMAC) treatment of patients with Kellgren–Lawrence (K–L) grades II–III knee osteoarthritis (OA) over a minimum follow-up period of 6 months. This study retrospectively evaluated data from 231 patients (285 knees) with knee OA treated with BMAC articular injection at a single center from August 2023 to October 2023. The inclusion criteria were a longstanding knee pain unresponsive to conservative treatments for at least 6 weeks with K–L grades II–III OA. The exclusion criteria were age of <40 years or >80 years, previous knee surgery, rheumatological or other systemic disease, malignancy, uncontrolled diabetes mellitus, or infections. Bone marrow was aspirated from the anterior iliac crest and concentrated by the single-spin centrifugation technique. The visual analog scale (VAS) pain score and Knee Society Score were used to evaluate the clinical outcomes and complications associated with harvest and injection sites were evaluated. The mean follow-up period was 7.2 months (range: 6–8 months). The pretreatment VAS pain score decreased from 4.3 to 0.4 points at the final follow-up (*p* < 0.05). Pretreatment Knee Society knee and function scores were improved from 86.9 to 98.1 (*p* < 0.05) and from 68.4 to 83.3 points (*p* < 0.05), respectively. A total of 15 complications (5.3%, 15/285) were observed, including 3 hematomas, 2 numbness, 2 contact dermatitis, and 1 superficial infection in the harvest site and 4 mild and moderate swelling and 3 severe swelling and pain in the injection site. BMAC is a reliable and effective treatment for patients with K–L grades II–III knee OA, but the orthopedic surgeon should consider that bleeding tendency by heparin causes severe joint swelling and pain after intra-articular knee injection.

## 1. Introduction

Knee osteoarthritis (OA) is a chronic degenerative disease-causing irreversible deterioration and articular cartilage loss in the entire joint and resulting in a poor quality of life [1]. Knee OA is the most prevalent degenerative joint disease that affects 3.8–16% of the population [2,3,4]. Current nonsurgical strategies, such as weight loss, physical therapy, medications, and intra-articular injections, are limited and mainly focus on diminishing symptoms and disability rather than curing OA itself [5]. Total knee arthroplasty (TKA), which is encumbered by several complications, is the definitive treatment for end-stage knee OA [6]. Thus, novel bioregenerative therapies, such as mesenchymal stem cells (MSCs), are an area of growing interest to delay or prevent TKA requirements.

MSCs, obtained from various sources, are multipotent cells that demonstrate the ability to repair compromised articular cartilage and slow knee OA progression [7,8,9]. MSCs have been applied in clinical practice since 1995, and bone marrow aspirate concentrate (BMAC) represents a safe and reliable source of MSCs. The advantage of BMAC is its composition, including MSCs, hematopoietic precursors, monocytes, endothelial cells, growth factors, and cytokines [10]. Clinically, BMAC has exhibited promise in cartilage repair and regeneration in knee OA [8,9].

Intra-articular injection using BMAC is one of the bioregenerative therapies that use stem cell delivery that is currently approved by the Ministry of Health and Welfare in Korea and is increasingly used in treating knee OA. BMAC and MSCs have demonstrated promising results in cartilage repair and regeneration, pain, and function at short-term follow-up in knee OA [8,9,11]. However, controversy remains in the clinical evidence and the indication of BMAC for knee OA [12,13,14]. Furthermore, only a few studies discuss the complications of harvest and injection sites associated with BMAC.

This study aimed to determine the effectiveness and potential complications on the harvest site and knee of BMAC treatment in patients with Kellgren–Lawrence (K–L) grades II–III knee OA over a minimum 6-month follow-up period.

## 2. Materials and Methods

The Institutional Review Board of our hospital, which waived the requirement for informed consent, approved the design and protocol of this retrospective study.

This study retrospectively evaluated a consecutive series of 239 patients who received BMAC injections (297 knees) for symptomatic knee OA from August 2023 to October 2023. A total of 58 patients underwent BMAC injections in both knees. Inclusion criteria were a longstanding knee pain unresponsive to conservative treatments for at least 6 weeks with K–L grades [15] II–III OA. Exclusion criteria were K–L grade I or IV OA, age of <40 years or >80 years, previous knee surgery, rheumatological or other systemic disease, malignancy, uncontrolled diabetes mellitus, or infections. This study excluded 8 (12 knees) of the 239 patients (297 knees) due to follow-up loss. The final cohort consisted of 172 females (210 knees) and 59 males (75 knees). Demographic data, including sex, age, body mass index, K–L grades, pretreatment status assessed by the visual analog scale (VAS) pain score, and pretreatment Knee Society Score (KSS) [16], were obtained by reviewing medical records (Table 1). The mean follow-up period was 7.2 months (range: 6–8 months).

Patient charts were reviewed to determine any complications associated with harvest and injection sites after BMAC treatments. Clinical outcomes were assessed using the VAS (an 11-scale pain rating from 0 for no pain to 10 for the worst possible pain) and the KSS system [16]. All the patients were monitored using the same standard therapy. The patients were allowed to bear full weight and instructed to return to light activity as tolerated, avoiding oral painkillers after BMAC injections. There were no other therapeutic interventions (bracing, physical therapy, etc.). The patients underwent clinical and radiographic follow-up examinations at 2 weeks, 1, 3, 6, 9, and 12 months post-treatment. Any patients who did not return for their scheduled visits were contacted by telephone during these follow-up evaluations. Two nurses and one private doctor determined and visited nonresponders.

The pre- and post-treatment scores were compared using the paired t-test for statistical analysis. An analysis of variance (ANOVA) was performed to detect the treatment effects on the measured variables. Statistical Package for the Social Sciences version 18.0 (Chicago, IL, USA) was used for analyses. All reported *p*-values were two-sided, and *p*-values of <0.05 were considered statistically significant.

### BMAC Harvest and Injection Procedure

The patient was positioned supine on the operating table. The harvest was conducted in the ipsilateral iliac crest of the knee to be injected. A bump was positioned under the buttock to help expose the iliac crest. The area around the iliac crest was prepped and draped in a standard fashion with betadine and chlorhexidine.

**Figure 1:**
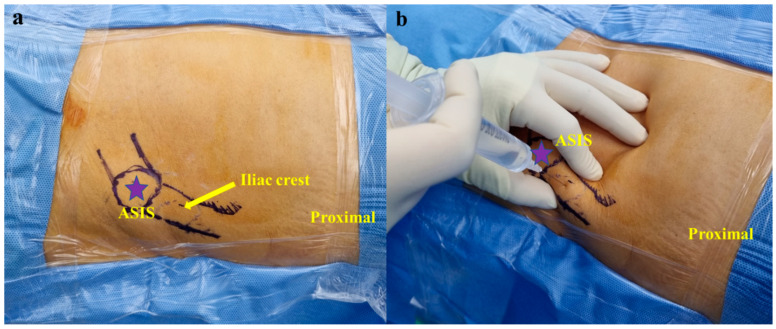
(**a**) Left anterior superior iliac spine (ASIS) and iliac crest. (**b**) 2% lidocaine is placed around the harvest site (2–3 cm proximal to the ASIS).

**Figure 2:**
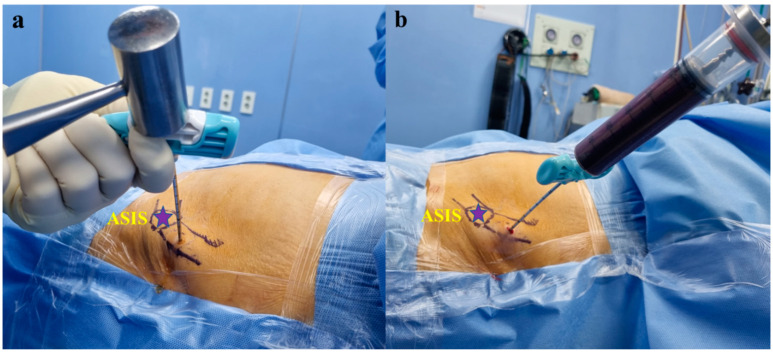
(**a**) The BMAC harvest needle is advanced approximately 2 cm (2 divisions on scale) from the midpoint of the iliac crest between the inner and outer cortices and in a trajectory parallel to the iliac crest. (**b**) The bone marrow is collected using a heparin-coated syringe with (heparin of 1 cc and normal saline of 4 cc) for a total of 60 cc.

**Figure 3:**
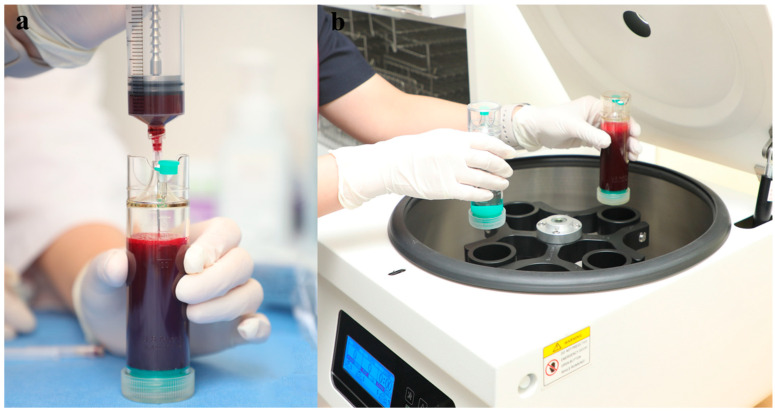
(**a**) Harvested bone marrow is transferred into a disposable sterile container. (**b**) Concentrate by single-spin centrifugation technique at 4000 RPMs for 12 min to harvest BMAC.

The anterior superior iliac spine (ASIS) was palpated and 2% lidocaine was applied around the harvest site (2–3 cm proximal to the ASIS) (Figure 1). A 0.5 cm stab incision with a No. 11 scalpel was performed over the harvest site. The BMAC harvest needle (PRO-BMC, Goodmorning Bio, Incheon, Republic of Korea) with a sharp trocar was then introduced percutaneously through the skin. The inner and outer cortices of the iliac crest were palpated with the trocar. The BMAC harvest needle was advanced approximately 2 cm (2 divisions on the scale) from the midpoint of the iliac crest between the inner and outer cortices, and in a trajectory parallel to the iliac crest (Figure 2a). The sharp trocar was then pulled out and bone marrow was collected using a heparin-coated syringe (heparin of 1 cc and normal saline of 4 cc) for a total of 60 cc (Figure 2b). The harvest site was sutured and dressed, and compression was applied for 5 min to stop bleeding. Harvested bone marrow was transferred into a disposable sterile container (PRO-BMC Kit) and concentrated by the single-spin centrifugation technique for 12 min at 4000 RPMs obtaining BMAC (Figure 3). Plasma and red blood cell components were removed and BMAC of approximately 6 cc was obtained for injection.

**Figure 4:**
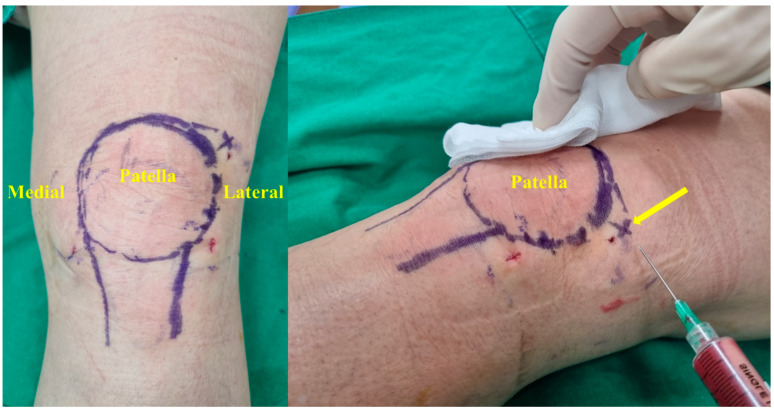
BMAC is injected into the patient’s affected knee through a superolateral margin (arrow) of the patella.

BMAC of 6 cc was injected in the patient’s affected knee through a superolateral margin of the patella (Figure 4). The knee was flexed and extended after the intra-articular injection to diffuse the BMAC in the knee joint. The patients were allowed full-weight bearing and instructed to return to light activity as tolerated. The procedure was repeated on the other side in the same method in patients with bilateral knee OA.

## 3. Results

The study included 285 BMAC procedures with 231 patients. The mean VAS pain score decreased from 4.3 points pretreatment to 0.4 points at the final follow-up (*p* < 0.05). The mean Knee Society knee and function scores improved from 86.9 to 98.1 points (*p* < 0.05) and 68.4 to 83.3 points from pretreatment to the final follow-up, respectively (*p* < 0.05) (Table 2). The patients with K–L grades II and III were assessed separately as follows in Table 2. ANOVA was performed to determine the difference in the VAS pain score, Knee Society knee score, and Knee Society function score before treatment and after 1, 3, and 6 months of treatment (Table 2).

We defined a meaningful complication as any symptom lasting ≥3 days or severe pain at the harvest site and knee after the BMAC procedure. A total of 15 meaningful complications were found at the harvest and injection sites (5.3%, 15/285). Hematoma was developed in three harvest sites. All hematomas were conservatively treated, achieving eventual resolution and no sequelae. Numbness occurred at two harvest sites, all of which recovered within 3 months. Two cases of contact dermatitis and one superficial infection occurred at the harvest site, which all recovered within 1 month with medication. Major adverse effects, such as infection and iliac crest fracture, did not occur at any of the harvest sites. Four cases of mild and moderate swelling occurred in the knee injection site, all of which disappeared within 2 weeks without any special measures. Three cases of severe swelling and pain occurred in the knee, and all were treated with decompression via joint aspiration and compression dressing 1 day after the BMAC procedure. None of the injection sites had an infection.

## 4. Discussion

Several published studies supported the efficacy and safety of BMAC treatment in knee OA [8,9,11,17,18]. Additionally, some studies revealed potential complications of the iliac crest as harvest sites are rare, and no serious adverse events occurred after the BMAC procedure [17,18]. However, most of the studies exhibited no clear definition of complications associated with the BMAC procedure and focused mostly on the effectiveness of BMAC. Our study reports the most important result that the use of BMAC to treat patients with K–L grades II–III knee OA is effective, but complications associated with the BMAC procedure are not particularly rare.

Our study results are similar to previously published reports of BMAC treatment in knee OA as they had pain relief and function improvement. Kim et al. [9] conducted a study on 75 knees affected by OA treated by a single BMAC articular injection and revealed the efficacy of this therapy in early to moderate knee OA. Chahal et al. [19] demonstrated the effectiveness of a single BMAC articular injection in knee OA, with an overall improvement in symptoms and pain as well as synovial inflammation reduction. Our study revealed that BMAC articular injection is effective in improving pain and function in patients with moderate knee OA at a minimum 6-month follow-up. Additionally, longer follow-up is warranted to validate these results.

The 5.3% complication rate associated with the BMAC procedure is significant, considering the increasing use of intra-articular injection using BMAC in treating knee OA. BMAC-associated complications occur both at the site where the autogenous bone marrow is harvested and at the knee where the MSCs are injected. A few studies focus on BMAC-associated complications of the harvest site and knee. The current study reports the largest clinical data series on this topic. BMAC can be harvested from multiple locations, including the iliac crest, proximal or distal tibia, and calcaneus. Several studies revealed that the iliac crest possesses the largest number of osteogenic progenitor cells [20,21]. Therefore, we selected the iliac crest as our harvest site. The iliac crest is a well-known standard harvest site for autologous bone grafting, and complications associated with donor-site harvesting from it are well documented, including chronic pain, infection, hematoma, lateral femoral cutaneous nerve injury, neuroma, chronic numbness, fracture, and hernia [22]. Potential complications, including chronic pain, infection, hematoma, lateral femoral cutaneous nerve injury, neuroma formation, persistent numbness, harvest site fracture, and incisional hernia, remain despite harvesting BMAC from the iliac crest through a percutaneous procedure. Only minor, mostly self-limiting complications have occurred in our study despite concerns about various complications. Hematoma occurred in three cases and was the most prevalent complication in the harvest sites. All hematomas were conservatively treated and achieved eventual resolution and no sequelae, but applying pressure to the puncture site for 10 min after the procedure is recommended. Two cases of numbness are thought to be due to lateral femoral cutaneous nerve injury or local anesthesia. Two cases of contact dermatitis were not reported in the other papers, presumably caused by the bandages, which resolved 1 month postprocedure. One superficial infection occurred in a patient with obesity, probably caused by the skin overlapping that continues to irritate the wound, which was resolved with oral antibiotics. Our study revealed no major adverse effects, such as bone infection and iliac crest fracture, on the harvest sites.

Intra-articular knee injections are a relatively safe procedure. However, certain risks and complications, such as pain, swelling, injection site infection, joint lining inflammation, septic arthritis, or osteonecrosis, may occur depending on the substance injected into the joint cavity. The most prevalently used injection therapies include corticosteroids, platelet-rich plasma, and viscosupplementation with substances such as hyaluronic acid. Additionally, a study reported complications in the knee joint [23]. However, the literature evidence regarding studies investigating potential adverse joint events after treatment with intra-articular injection of BMAC remains relatively lacking. Our study revealed mild and moderate joint swelling as the most prevalent adverse events, which resolved within two weeks without any special measures. This may be related to the autologous biological property of MSCs [24]. However, three cases (1.1%, 3/285) exhibited severe knee pain and swelling postprocedure. All patients underwent joint aspiration due to severe pain and swelling, and the joint fluid was bloody. The symptoms disappeared 2–3 days after knee joint aspiration and compression. It is assumed that the bleeding tendency may have been caused by the effect of heparin mixed into the BMAC when bone marrow is collected using a heparin-coated syringe. Therefore, the orthopedic surgeon should consider that the bleeding tendency of heparin causes severe joint swelling and pain after intra-articular knee injection.

This study had several limitations. First, this study was not a single-surgeon series; however, all surgeons used the same technique and consistent peritreatment protocols. Second, this was a single-center study; thus, the generalizability of the results and complications are limited. Third, the follow-up period was short. However, the six-month follow-up period was sufficient to determine complications associated with the BMAC procedure. The strength of this study is that it focuses on BMAC-associated complications.

## 5. Conclusions

This study showed that BMAC treatment in patients with K–L grades II–III knee OA provides reliable and effective clinical outcomes over a 6-month period, but BMAC-associated complications are not rare. In particular, the orthopedic surgeon should consider that the bleeding tendency of heparin can cause severe joint swelling and pain after intra-articular knee injection. Therefore, it is essential to consider the possibility of complications following BMAC treatment at all times, necessitating close observation for several days after the procedure.

## Figures and Tables

**Table 1:** Patient demographics.

	Final Cohort
Number of patients (knees)	231 (285)
Male:female	59 (75):172 (210)
Age (years) (mean ± SD)	64.2 ± 7.5
Body mass index	24.9 ± 3.2
K–L grades	
II	86
III	199
Pretreatment	
VAS pain score	4.3 ± 4.2
Pretreatment	
Knee Society knee score	86.9 ± 7.9
Function score	68.4 ± 14.2

SD: standard deviation, K–L: Kellgren–Lawrence, and VAS: visual analog scale.

**Table 2:** Clinical results at each follow-up.

	1. Pretreatment	2. PTD 1 Month	3. PTD 3 Months	4. PTD 6 Months	*p*-Value	Post Hoc
VAS pain score						
All	4.3 ± 4.2	1.5 ± 1.1	0.8 ±1.0	0.4 ± 0.6	<0.05	1 > 2 > 3 = 4
K–L grade II	4.0 ± 1.7	1.3 ± 1.0	0.5 ± 0.6	0.2 ± 0.4	<0.05	
K–L grade III	4.6 ± 5.5	1.7 ± 1.1	1.1 ± 1.1	0.5 ± 0.7	<0.05	
KS knee score						
All	86.9 ± 7.9	94.2 ± 3.8	96.7 ± 3.5	98.1 ± 2.7	<0.05	4 > 3 > 2 > 1
K–L grade II	87.1 ± 8.9	94.8 ± 3.7	97.8 ± 2.5	98.6 ± 2.2	<0.05	
K–L grade III	86.8 ± 7.0	93.8 ± 3.9	95.7 ± 4.0	97.7 ± 3.0	<0.05	
KS function score						
All	68.4 ± 14.2	74.9 ± 15.4	82.8 ± 13.6	83.3 ± 17.1	<0.05	4 > 3 > 2 > 1
K–L grade II	68.8 ± 15.4	76.1 ± 15.4	84.9 ± 11.9	85.7 ± 15.1	<0.05	
K–L grade III	68.0 ± 13.1	74.0 ± 15.4	81.0 ± 14.6	81.3 ± 18.5	<0.05	

PTD: post-treatment, VAS: visual analog scale, K–L: Kellgren–Lawrence, KS: Knee Society.

## Data Availability

The data presented in this study are available from the corresponding authors upon request. The data are not publicly available due to privacy reasons.
